# Synthesis and characterization of Co_x_Fe_1−x_Fe_2_O_4_ nanoparticles by anionic, cationic, and non-ionic surfactant templates via co-precipitation

**DOI:** 10.1038/s41598-022-08709-9

**Published:** 2022-03-17

**Authors:** Kittipon Sangsuriyonk, Nophawan Paradee, Kornkanok Rotjanasuworapong, Anuvat Sirivat

**Affiliations:** 1 Conductive and Electroactive Polymers Research Unit, The Petroleum and Petrochemical College, Chulalongkorn University, Bangkok, 10330 Thailand; 2 Department of Chemistry, Faculty of Science, King Mongkut’s University of Technology Thonburi, Bangkok, 10140 Thailand

**Keywords:** Materials science, Nanoscience and technology

## Abstract

The cobalt ferrite nanoparticles (Co_x_Fe_1−x_Fe_2_O_4_) were synthesized by the surfactant templated co-precipitation method using various surfactants namely sodium dodecyl sulfate (SDS), hexadecyltrimethylammonium bromide (CTAB), and Tween20. Under the substitution, the Co_x_Fe_1−x_Fe_2_O_4_ particles were synthesized at various Co^2+^ and Fe^2+^ mole ratios (x = 1, 0.6, 0.2, and 0) with the SDS. The cobalt ferrite nanoparticles were characterized for their morphology, structure, magnetic, and electrical properties. All Co_x_Fe_1−x_Fe_2_O_4_ nanoparticles showed the nanoparticle sizes varying from 16 to 43 nm. In the synthesis of CoFe_2_O_4_, the SDS template provided the smallest particle size, whereas the saturated magnetization (M_s_) of CoFe_2_O_4_ was reduced by using CTAB, SDS, and Tween20. For the Co_x_Fe_1−x_Fe_2_O_4_ as synthesized by the SDS template at 1.2 CMC, the M_s_ increased with increasing Fe^2+^ mole ratio. The highest M_s_ of 100.4 emu/g was obtained from the Fe_3_O_4_ using the SDS template. The Fe_3_O_4_ nanoparticle is potential to be used in various actuator and biomedical devices.

## Introduction

Magnetic nanoparticles have been widely investigated for many applications such as magnetic fluid^1^, catalysis^2^, magnetic resonance imaging (MRI)^3^, proton exchange membrane^4^, actuator^5^, hyperthermia^6^, and drug delivery^7^. Substitution of various divalent cations (M^2+^) namely Co^2+^, Mn^2+^, Zn^2+^, Mg^2+^, and Ni^2+^ into ferrite nanoparticles can significantly alter their magnetic properties^8^. Among the ferrites magnetic nanoparticles with the spinel structures, CoFe_2_O_4_ provides the notable properties namely: chemical stability, high coercivity (H_c_), and high Curie temperature^9^. Moreover, CoFe_2_O_4_ possesses a good anisotropic property as the Co^2+^ substitution provides a higher degree of anisotropy relative to Fe^2+^ and Fe^3+^^10^. However, the bulk saturated magnetization (M_s_) of CoFe_2_O_4_ (80 emu/g) obtained so far is still lower than Fe_3_O_4_ (presently at ~ 89 emu/g)^11^.

The shape, size, and properties of magnetic particles are generally dictated by the synthesis method^12–14^. There are various methods to synthesize magnetic nanoparticles such as hydrothermal^15^, sol-gel^16^, micro-emulsion^17^, thermal decomposition^18^, and co-precipitation^19^. Among these techniques, the co-precipitation is a simple method as it is inexpensive, with a short reaction time and a lower reaction temperature. The important factors namely the reaction temperature, stirring speed, and pH of the reactant are essential in controlling the particle shape and size as related to the particle nucleation and growth rates. Ideally, the nucleation rate should be higher than the growth rate to obtain smaller particles.

Alternatively, the particle shape and size can be manipulated by using surface-active agents, namely surfactants, because of their electrostatic repulsion and steric hindrance properties. In particular, the surfactant could reduce the agglomeration of the magnetic nanoparticles from the magnetic interaction and with high surface reactivity. Vadivel et al. used sodium dodecyl sulfate (SDS) as the surfactant for the co-precipitation synthesis of CoFe_2_O_4_ under various SDS concentrations. SDS improved the particles size distribution and magnetic property of CoFe_2_O_4_^20^.

Nanomagnetic particles (NMPs) have been utilized in various applications, in particular actuators^21–25^ and biomedical devices^26–30^. The important and required features of NMP for these applications are the high magnetization, superparamagnetic behavior, and non-toxicity towards human.

In this work, the effect of surfactant types, namely sodium dodecyl sulfate (SDS), hexadecyltrimethylammonium bromide (CTAB), and Tween20 as anionic, cationic, and non-ionic surfactants, were investigated on the synthesis of Co_x_Fe_1−x_Fe_2_O_4_ with 0 ≤ x ≤ 1 and on the resultant magnetic properties. It will be shown that SDS was the most suitable surfactant for the synthesis of CoFe_2_O_4_ with the nanoparticle size of 16 ± 3 nm, whereas the highest magnetization as obtained from the Fe_3_O_4_ by the SDS template was as high as 100.41 emu/g with the superparamagnetic behavior. The synthesized Fe_3_O_4_ particle possesses magnetic properties which are potential to be used in various actuator and biomedical devices.

## Methods

### Materials

Iron (III) chloride (99% purity, Sigma Aldrish), cobalt (II) chloride (AR grade, Merck), and iron (II) sulfate heptahydrate (99% purity, Univar) were used as the precursors. Sodium dodecyl sulfate, SDS, (98.5% purity, Sigma Aldrich), hexadecyltrimethylammonium bromide, CTAB, (96% purity, Sigma Aldrich), and Tween20 (AR grade, Sigma Aldrich) were the surfactants used. Sodium hydroxide, NaOH (AR grade, Univar) was utilized as a precipitating agent.

### Synthesis of CoFe_2_O_4_ magnetic nanoparticles by surfactant assisted co-precipitation under various surfactant types

Metal precursors including iron (III) chloride (Fe^3+^), and cobalt (II) chloride (Co^2+^) with the Fe^3+^: Co^2+^ molar ratio of 0.10: 0.05 (0.81 g: 0.33 g) were put in 25 ml deionized water. The metal ion solution was separately mixed with 25 ml of various surfactant solutions namely: SDS (8.2 mM^31^, 0.12 g), CTAB (0.92 mM^32^, 0.003 g), and Tween 20 (0.06 mM^33^, 0.02 g) at their critical micelle concentrations (CMC): To obtain the CMC data, the surfactant solution in water was tested at 25 °C^31–33^. Each surfactant was dissolved in the deionized water and was stirred for 30 min to form micelles before adding the metal ions at room temperature. The mixture solution was continuously stirred at room temperature for 30 min. After that, 3 M NaOH solution (15 ml) was added dropwise and then continuously stirred for 4 h at 80 °C. The obtained dark precipitate was washed with water and ethanol to eliminate the remaining surfactant, and then dried at 80 °C for 24 h. The synthesized CoFe_2_O_4_ by SDS, CTAB, and Tween20 as the surfactants and no surfactant are coded as CoFe_2_O_4__SDS_1CMC, CoFe_2_O_4__CTAB_1CMC, CoFe_2_O_4__Tween20_1CMC, and CoFe_2_O_4__Bare, respectively.

### Synthesis of Co_x_Fe_1−x_Fe_2_O_4_ magnetic nanoparticles by surfactant assisted co-precipitation under various molar ratio of Co^2+^ and Fe^2+^

CoFe_2_O_4_, Co_0.6_Fe_0.4_Fe_2_O_4_, Co_0.2_Fe_0.8_Fe_2_O_4_, and Fe_3_O_4_ were synthesized with the metal precursors including iron (III) chloride (Fe^3+^), cobalt (II) chloride (Co^2+^), and iron (II) sulfate (Fe^2+^) at the Fe^3+^: Co^2+^: Fe^2+^ molar ratios of 0.10: 0.05: 0.00 (0.81 g: 0.33 g: –), 0.10: 0.03: 0.02 (0.81 g, 0.26 g, 0.14 g), 0.10: 0.01: 0.04 (0.811 g: 0.07 g: 0.56 g), and 0.10: 0.00: 0.05 (0.811 g: –: 0.70 g), where they were dissolved in 25 ml deionized water. The SDS (10 mM, 0. 14 g) was dissolved in 25 ml deionized water for 30 min and then each metal precursor solution was put in the SDS solution and stirred at room temperature for 30 min to obtain a homogeneous solution. After that, 3 M NaOH solution (15 ml) was added and then continuously stirred for 4 h at 80 °C. The obtained dark precipitate was washed with water and ethanol to eliminate the remaining surfactant and then dried at 80 °C for 24 h. The synthesized CoFe_2_O_4_, Co_0.6_Fe_0.4_Fe_2_O_4_, Co_0.2_Fe_0.8_Fe_2_O_4_, and Fe_3_O_4_ are coded as CoFe_2_O_4__SDS_1.2CMC, Co_0.6_Fe_0.4_Fe_2_O_4__SDS_1.2CMC, Co_0.2_Fe_0.8_Fe_2_O_4__SDS_1.2CMC, and Fe_3_O_4__SDS_1.2CMC, respectively.

### Cobalt ferrite nanoparticles characterization

A wide angle X-ray diffractometer, XRD, (Rigaku, SmartLab) was utilized to investigate the crystalline structures of the magnetic nanoparticles. The CuK-alpha radiation source was employed at 40 kV/30 mA using the K-beta filter to eliminate interference peaks. The diffractometer was fitted with the Bragg–Brentano geometry, the graphite monochromator and the diffracted beam, and operated at a scan rate of 2°/min and a scan step of 0.02°. Each sample was dried and grinded to obtain a fine powder. The sample was put into a mold and then compressed by a hydraulic machine.

A Fourier transform infrared spectrometer, FT-IR, (Nicolet, iS5) was employed to measure spectra of the magnetic nanoparticles using potassium bromide (KBr) as the background material. To prepare a sample, a small amount of sample powder was mixed and grinded with KBr. The mixture powder was put into a mold and then compressed by a hydraulic pressure machine for 15 s. The spectra were measured in the wavenumber range of 650 cm^−1^ to 4000 cm^−1^.

A scanning electron microscope, SEM, (Hitachi, S-4800) was used to study the morphological structure and to measure the magnetic nanoparticle sizes. Each sample was coated with a thin layer of platinum. The images were obtained at the acceleration voltage of 5 kV and at the magnifications of 100,000 and 150,000.

An electron dispersive spectrometer, EDS, (FE-SEM Hitachi, S-4800) was used to determine the atomic percentages of the cobalt ferrite nanoparticles. Each sample was coated with a thin layer of platinum.

An X-ray photoelectron spectroscope, XPS, (Kratos, Axis Ultra DLD) was employed to determine the atomic percentages of Co_x_Fe_1−x_Fe_2_O_4_ using the monochromatized Al K. Each sample was distributed on a carbon tape on the sample holder, and a copper grid was used as the reference for the elemental analysis.

A vibrating sample magnetometer, VSM, (LakeShore, Series 7400 model 7404) was employed to measure the saturated magnetization (M_s_), and coercivity (H_c_) of the cobalt ferrite nanoparticles. The measurements were taken under a magnetic field strength of 10,000 Gauss at room temperature, with 80 points/loop and with a scan speed of 10 s/point.

## Results and discussion

### Cobalt ferrite synthesis and characterization

**Figure 1:**
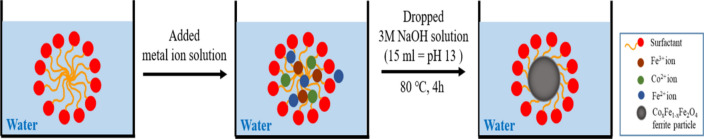
Surfactant assisted co-precipitation for synthesis of Co_x_Fe_1−x_Fe_2_O_4_.

The synthesis scheme is shown in Fig. 1. After the complete micelle formation at equal or above the critical micelle concentration (CMC), the metal ions (Fe^3+^, Fe^2+^, and Co^2+^) were added into the surfactant solution. The metal ions were stabilized with the spherical micelles of surfactant by the interaction between the polar groups of the surfactants and the metal cation precursors^34,35^. The synthesis reaction was carried out by adding NaOH (at the pH of 13) for 4 h under the nitrogen atmosphere to prevent the oxidation of ferrous ions (Fe^2+^) to ferric ions (Fe^3+^) by the oxygen atmosphere. In the case of SDS as an anionic surfactant, it could stabilize the metal cations by the micelle formation via the interaction between the polar group of SO_4_^–2^ and the metal cations^35^. After the adding NaOH to precipitate the ferrite particle, the OH^–^ from NaOH interacted with the metal cations to form the hydroxide precipitant and the SDS interacted with the hydroxide precipitant on the surface. The co-precipitation reaction is shown in Eq. (1)^36^.

1 $$\begin{gathered} {\text{2Fe}}^{{{3} + }} + {\text{6OH}}^{ - } \to {\text{2Fe}}\left( {{\text{OH}}} \right)_{{3}} \hfill \\ {\text{xCo}}^{{{2} + }} + {\text{2OH}}^{ - } \to {\text{xCo}}\left( {{\text{OH}}} \right)_{{2}} \hfill \\{\text{(1-x)Fe}}^{{{2} + }} + {\text{2OH}}^{ - } \to \left( {{1} - {\text{x}}} \right){\text{Fe}}\left( {{\text{OH}}} \right)_{{2}} \hfill \\ {\text{xCo}}\left( {{\text{OH}}} \right)_{{2}} + \left( {{1} - {\text{x}}} \right){\text{Fe}}\left( {{\text{OH}}} \right)_{{2}} + {\text{ 2Fe}}\left( {{\text{OH}}} \right)_{{3}} \to {\text{Co}}_{{\text{x}}} {\text{Fe}}_{{{1} - {\text{x}}}} {\text{Fe}}_{{2}} {\text{O}}_{{4}} + {\text{4H}}_{{2}} {\text{O}} \hfill \\ \end{gathered}$$

**Figure 2:**
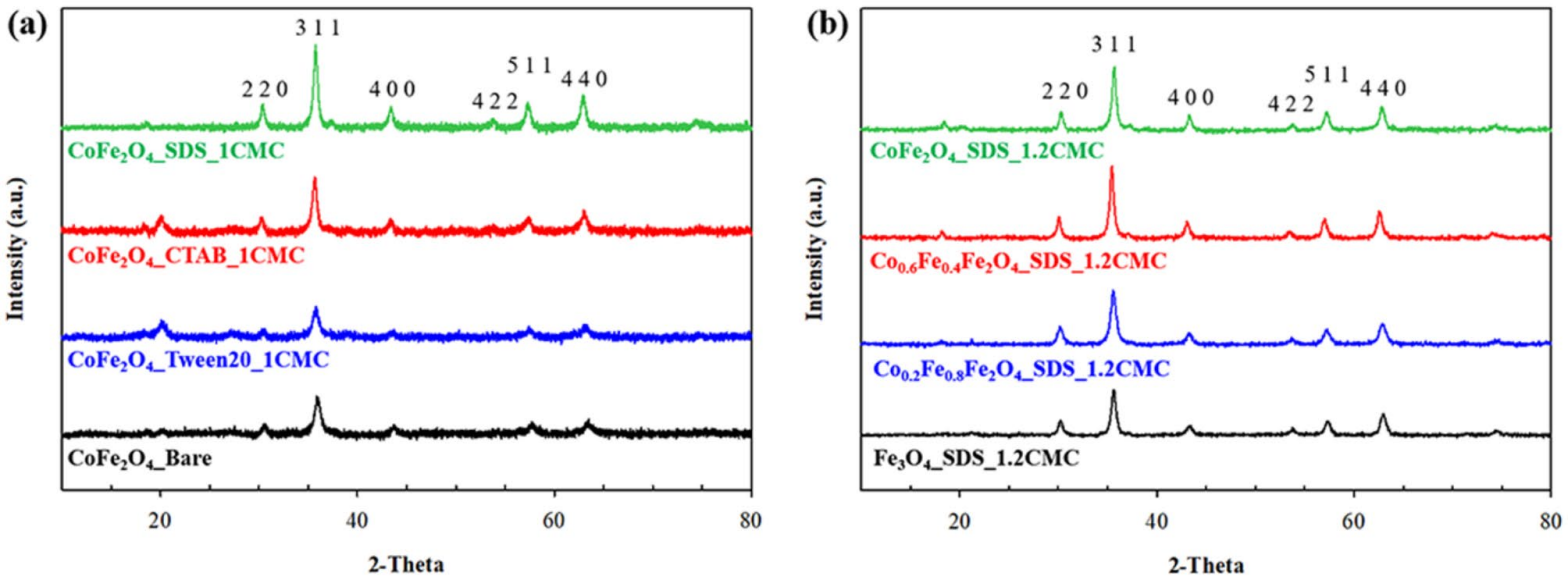
XRD patterns of CoFe_2_O_4_ and Co_x_Fe_1−x_Fe_2_O_4_: (**a**) under various surfactant types; (**b**) under various Fe^2+^ contents.

The crystalline structure of cobalt ferrite nanoparticles was characterized by the x-ray diffraction technique. Normally, magnetite nanoparticles are of a cubic spinel structure (AB_2_X) which composes of a divalent cation (A), a trivalent cation (B), and a divalent anion (X). The cations A and B occupy the octahedral or tetrahedral site of the spinel structure. Nevertheless, the ferrite nanoparticles can also form a reverse spinel structure, where the tetrahedral site is occupied by a trivalent cation and the octahedral site is occupied by a divalent cation and the remaining trivalent cation^37^. The XRD patterns of the CoFe_2_O_4_ as synthesized by SDS, CTAB, Tween20 and without surfactant are shown in Fig. 2a. The patterns of CoFe_2_O_4_ synthesized by all surfactants show the major characteristic peaks at (2 2 0), (3 1 1), (4 0 0), (4 2 2), (5 1 1), and (4 4 0) which reflect a cubic spinel structure^38^. Table 1 lists the calculated average crystallite sizes. The average crystallite size was calculated by using the (3 1 1) peak and Eq. (2):

2 $${\text{t}} = \frac{{{\text{k}}\uplambda }}{\upbeta \cos \theta }$$

**Table 1:** Co: Fe mole ratios, average crystallite sizes (t_311_), lattice constants (a), unit volume cells (V_cell_), hopping lengths for tetrahedral site (L_A_), hopping lengths for octahedral site (L_B_), particle sizes, and the Co: Fe atomic ratios from EDS and XPS of cobalt ferrite nanoparticles.

Sample	Co: Fe mole ratio	XRD					SEM	EDS	XPS
		Average crystallite size (t_311_) (nm)	Lattice constant (a) (Å)	Volume (V_cell_)	L_A_ (nm)	L_B_ (nm)	Particle size (nm)	Co: Fe atomic ratio	Co: Fe atomic ratio
CoFe_2_O_4__Bare	–	10.9	8.32	596.22	3.60	2.94	42 ± 8	–	–
CoFe_2_O_4__SDS_1CMC	–	15.9	8.34	579.05	3.61	2.95	16 ± 3	–	–
CoFe_2_O_4__CTAB_1CMC	–	12.5	8.34	579.68	3.61	2.95	20 ± 3	–	–
CoFe_2_O_4__Tween20_1CMC	–	9.21	8.32	597.60	3.60	2.94	21 ± 3	–	–
CoFe_2_O_4__SDS_1.2CMC	1:2	16.8	8.35	581.96	3.62	2.95	22 ± 3	1:1.8	1:1.9
Co_0.6_Fe_0.4_Fe_2_O_4__SDS_1.2CMC	1:4	18.7	8.41	596.18	3.64	2.98	24 ± 3	1:3.5	1:3.9
Co_0.2_Fe_0.8_Fe_2_O_4__SDS_1.2CMC	1:14	11.7	8.36	585.51	3.62	2.96	32 ± 4	1:12	1:14
Fe_3_O_4__SDS_1.2CMC	0:1	9.81	8.35	583.11	3.62	2.95	43 ± 8	0:1	0:1

where k is the dimensionless shape factor (k = 0.9), λ is the X-ray wavelength (CuKa = 1.5405 Å), β is the full width at the half maximum of diffraction peak (3 1 1), and θ is the angle of diffraction (2θ/2). The lattice constant (a) was calculated by using the (3 1 1) peak and Eq. (3):

3 $${\text{a}} = {\text{d}}\sqrt {{\text{h}}^{2} + {\text{k}}^{2} + {\text{l}}^{2} }$$

where d is the interplanar spacing, and (h l k) are the Miller indices. The volume unit cell was calculated by Eq. (4):

4 $${\text{V}}_{{{\text{cell}}}} = {\text{a}}^{{3}}$$

The hopping lengths for the tetrahedral site (L_A_) and octahedral site (L_B_) were calculated by Eqs. (5–6)^39^:

5 $${\text{L}}_{{\text{A}}} {\text{ = a}}\frac{\sqrt 3 }{4}$$

and

6 $${\text{L}}_{{\text{B}}} {\text{ = a}}\frac{\sqrt 2 }{4}$$

Table 1 also lists the calculated average crystallite sizes (t_311_), lattice constants (a), volumes (V_cell_), and hopping lengths (L_A_ and L_B_) of the cobalt ferrite nanoparticles synthesized. From the calculated crystallite sizes in Table 1, the CoFe_2_O_4_ synthesized using SDS as the surfactant possesses the largest crystallite size relative to other surfactant types which suggests that SDS improves the crystallinity of the CoFe_2_O_4_ as the negative charge of the SDS micelles stabilizes the cation and confine the space for crystallization^40^. However, the CoFe_2_O_4_ as synthesized by Tween20 and without surfactant show lower crystalline sizes than the CoFe_2_O_4_ with SDS or CTAB. This is because Tween20 (a non-ionic surfactant) and no surfactant could not stabilize the magnetic nanoparticles during the synthesis reaction resulting in a random crystallization.

The XRD patterns of Co_x_Fe_1−x_Fe_2_O_4_ are shown in Fig. 2b. From Table 1, the crystalline size of Co_x_Fe_1−x_Fe_2_O_4_ increases from 16.8 nm to 18.7 nm with x varying from 1.0 to 0.6, and then decreases to 9.81 nm at x equal to 0. This result suggests that the crystalline size decreases with increasing Fe^2+^ content or decreasing x from 0.6 to 0.0 due to the smaller grain size and the nanoparticle crystallinity^41^.

**Figure 3:**
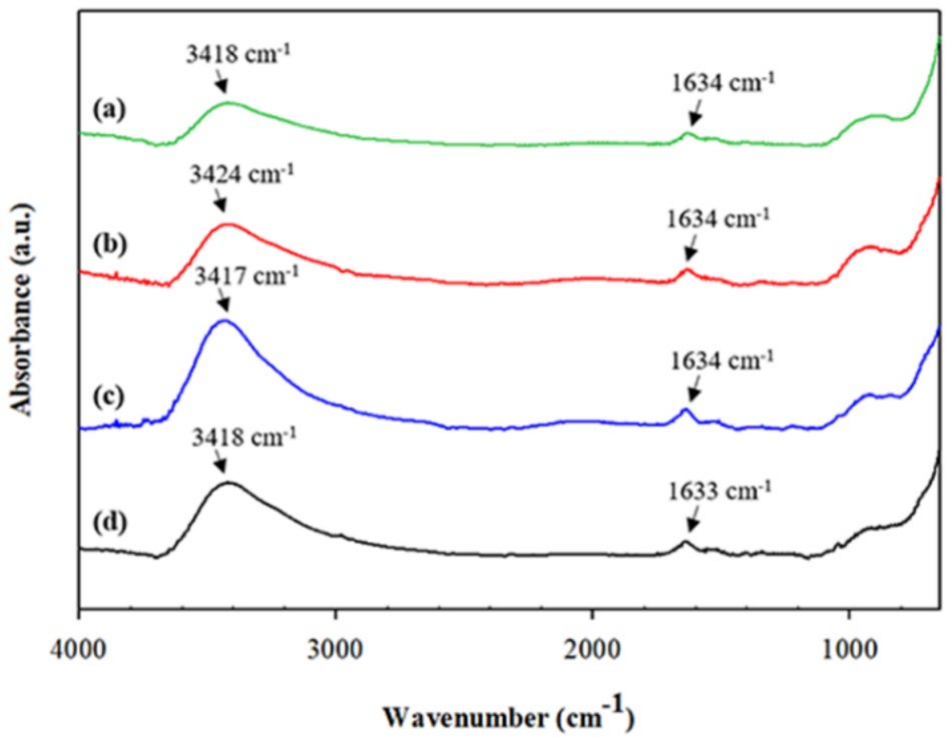
FT-IR spectra of the CoFe_2_O_4_ under various surfactant types: (**a**) CoFe_2_O_4__SDS_1CMC; (**b**) CoFe_2_O_4__CTAB_1CMC; (**c**) CoFe_2_O_4__Tween20_1CMC; and (**d**) CoFe_2_O_4__Bare.

**Figure 4:**
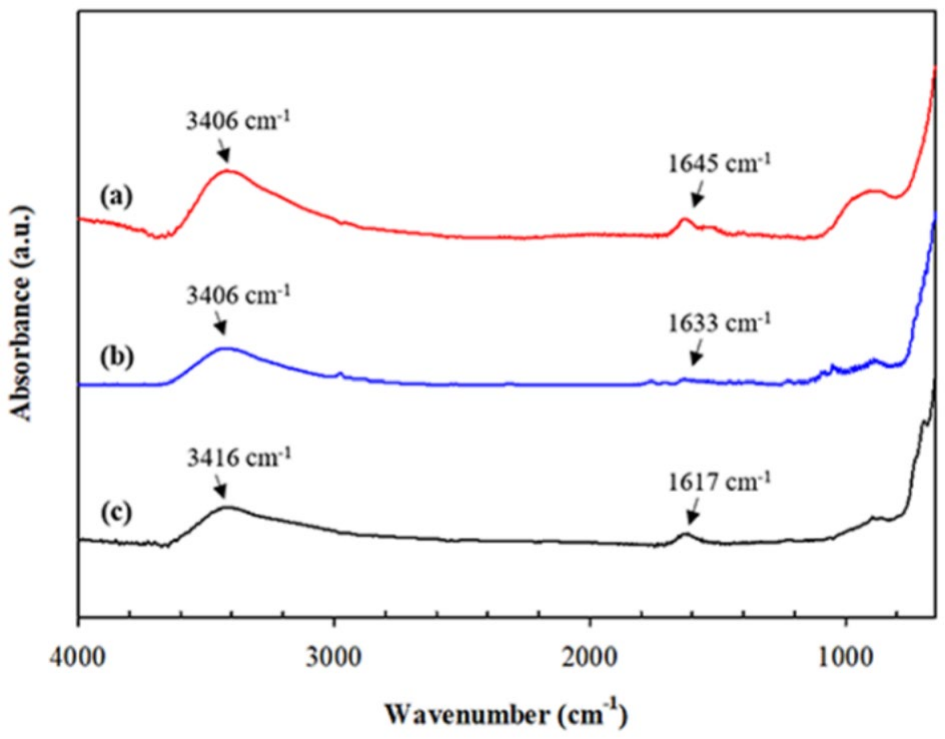
FT-IR spectra of the Co_x_Fe_1−x_Fe_2_O_4_: (**a**) CoFe_2_O_4__SDS_1.2CMC; (**b**) Co_0.2_Fe_0.8_Fe_2_O_4__SDS_1.2CMC; and (**c)** Fe_3_O_4__SDS_1.2CMC.

The FT-IR spectra of the synthesized cobalt ferrite magnetic nanoparticles under various surfactants and Co_x_Fe_1−x_Fe_2_O_4_ are shown in Fig. 3 and Fig. 4, respectively. All spectra show the identical peaks at around 1600 cm^−1^ and 3400 cm^−1^, corresponding the hydroxyl groups on the surface of the cobalt ferrite magnetic nanoparticles from the humidity^42^. In addition, there is no surfactant peak present which confirms the elimination of surfactants after washing out with water and ethanol. The SDS surfactant peaks should appear at 1113 cm^−1^, corresponding to the S–O stretching vibration; 1460 cm^−1^, corresponding to the C–O stretching; and 2923 and 2865 cm^−1^, corresponding to the C–H stretching vibration^35^.

The EDS technique was used to measure the atomic percentages corresponding to the Co: Fe mole ratio of the Co_x_Fe_1−x_Fe_2_O_4_ magnetic nanoparticles as shown in Table 1. The result shows that the EDS experimental Co: Fe mole ratios of Co_x_Fe_1−x_Fe_2_O_4_ are 1: 1.8, 1: 3.5, 1: 12 and 0: 1 for the CoFe_2_O_4_, Co_0.6_Fe_0.4_Fe_2_O_4_, and Co_0.2_Fe_0.8_Fe_2_O_4_, respectively. The calculated synthesis values of Co: Fe mole ratios are 1: 2, 1: 4, 1: 14, and 0: 1 respectively; thus, the EDS experimental values are quite close to the theoretical values.

**Figure 5:**
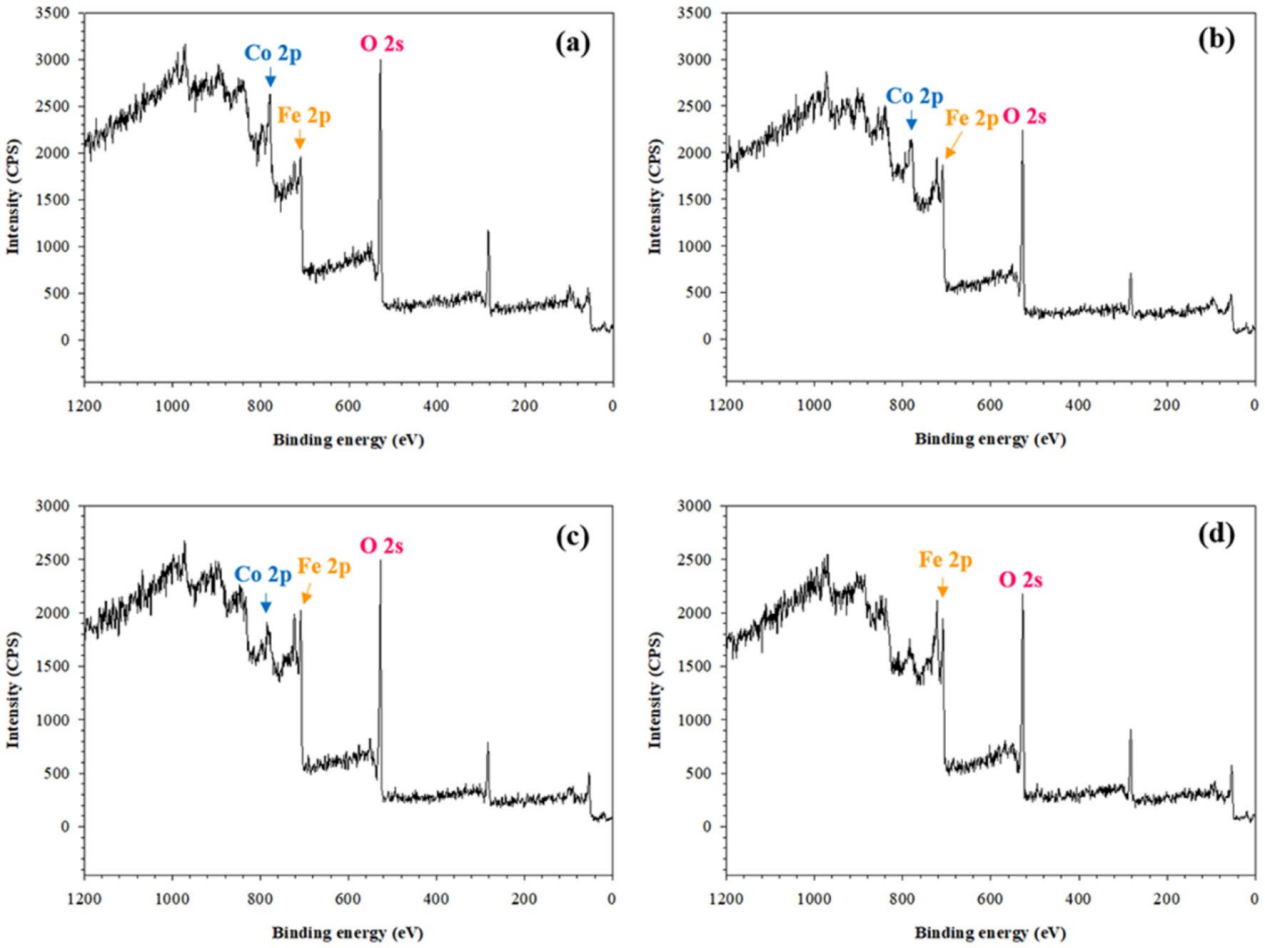
XPS spectra of the Co_x_Fe_1−x_Fe_2_O_4_: (**a**) CoFe_2_O_4__SDS_1.2CMC; (**b**) Co_0.6_Fe_0.4_Fe_2_O_4__SDS_1.2CMC; (**c**) Co_0.2_Fe_0.8_Fe_2_O_4__SDS_1.2CMC; and (**d**) Fe_3_O_4__SDS_1.2CMC.

The XPS technique was also used to confirm the Co: Fe mole ratio and the XPS spectra are shown in Fig. 5. The visible peaks can be observed at 778.3 eV, 706.7 eV, and 529.2 eV corresponding to the Co 2p, Fe 2p and O 1s respectively. The corresponding Co: Fe mole ratios of Co_x_Fe_1−x_Fe_2_O_4_ are 1: 1.9, 1: 3.9, 1: 14, and 0: 1, respectively. These mole ratio values from the EDS and XPS techniques are quite close thus confirming that the synthesized Co_x_Fe_1−x_Fe_2_O_4_ mole ratios match their theoretical stoichiometric values.

**Figure 6:**
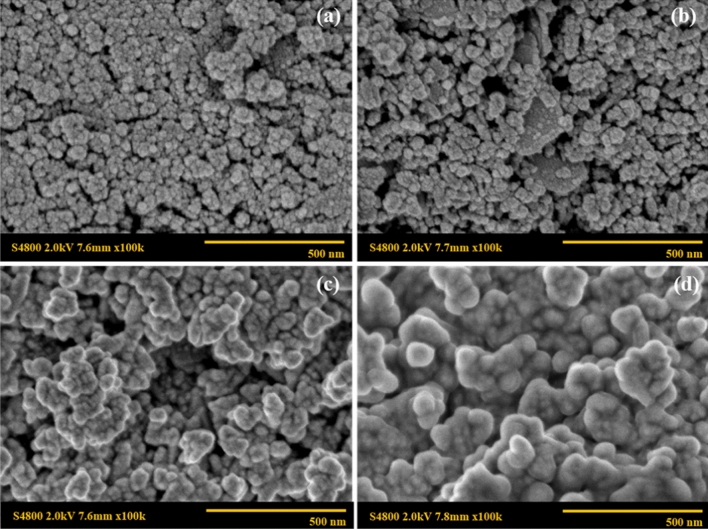
SEM images of CoFe_2_O_4_ under various surfactant types: (**a**) CoFe_2_O_4__ SDS_1CMC; (**b**) CoFe_2_O_4__CTAB_1CMC; (c) CoFe_2_O_4__Tween20_1CMC; and (**d**) CoFe_2_O_4__Bare.

**Figure 7:**
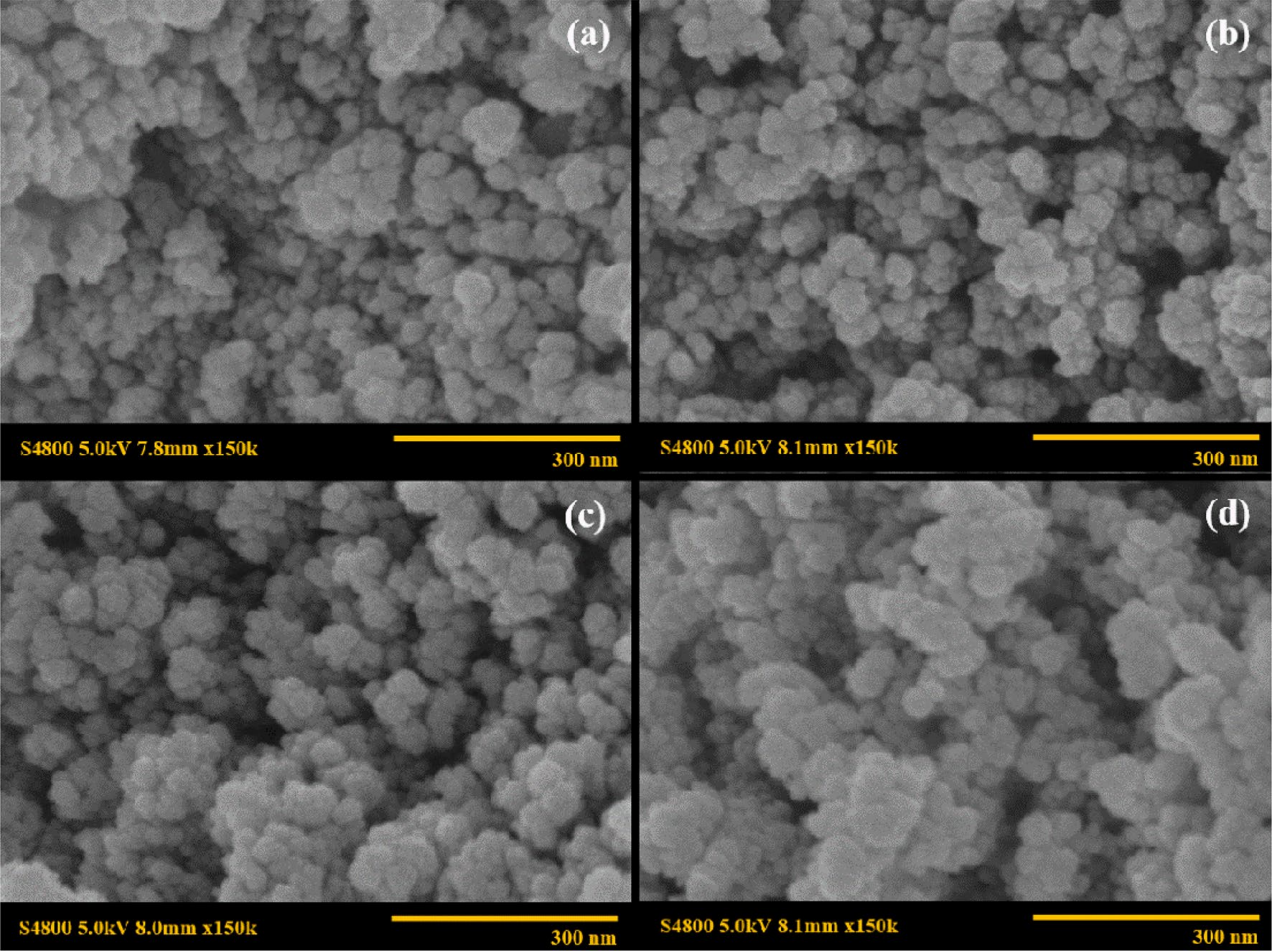
SEM images of Co_x_Fe_1−x_Fe_2_O_4_: (**a**) CoFe_2_O_4__SDS_1.2CMC; (**b**) Co_0.6_Fe_0.4_Fe_2_O_4__SDS_1.2CMC; (**c**) Co_0.2_Fe_0.8_Fe_2_O_4__SDS_1.2CMC; and (**d**) Fe_3_O_4__SDS_1.2CMC.

Morphology of the cobalt ferrite nanoparticles was investigated by the scanning electron microscope. In the case of CoFe_2_O_4_ under various surfactant types, the nearly spherical shapes of CoFe_2_O_4_ were obtained from all surfactants as shown in Fig. 6. The particle sizes of CoFe_2_O_4_ synthesized without surfactant, and with SDS, CTAB, and Tween20 are 42 nm, 16 nm, 20 nm, and 21 nm and, respectively. It appears that the particle size of cobalt ferrite nanoparticles as synthesized by the co-precipitation method was reduced by employing a surfactant because of the steric hindrance effect from the surfactant contributing to a slower nucleation and growth rate. Interestingly, SDS as an anionic surfactant provides the smaller particle size of 16 nm along with a narrow size distribution as the anion from SDS could stabilize the metal cations and the cobalt ferrite nanoparticles. For cases of CTAB and Tween20, the particle sizes are 20 nm and 21 nm, respectively, thus their sizes are comparable. However, the CoFe_2_O_4_ particle as synthesized by CTAB (cationic surfactant) tended to agglomerate and formed a larger flake, as shown in Fig. 6b. Figure 7 shows the nearly spherical shapes of CoFe_2_O_4_, Co_0.6_Fe_0.4_Fe_2_O_4_, Co_0.2_Fe_0.8_Fe_2_O_4_, and Fe_3_O_4_ with SDS at the surfactant concentration of 1.2 times the critical micelle concentration. The particle sizes are 22 nm, 24 nm, 32 nm, and 43 nm, respectively. For the different particle sizes of the Co_x_Fe_1−x_Fe_2_O_4_ ferrite particles, the particle sizes increased with increasing the Fe^2+^ substitution, indicating that the addition of Fe^2+^ effectively increases the crystal growth rate of Co_x_Fe_1−x_Fe_2_O_4_ with a larger particle size^43^. The smaller particles can be obtained when the nucleation rate is higher than the growth rate^44^.

### Magnetic property of cobalt ferrite nanoparticles

**Figure 8:**
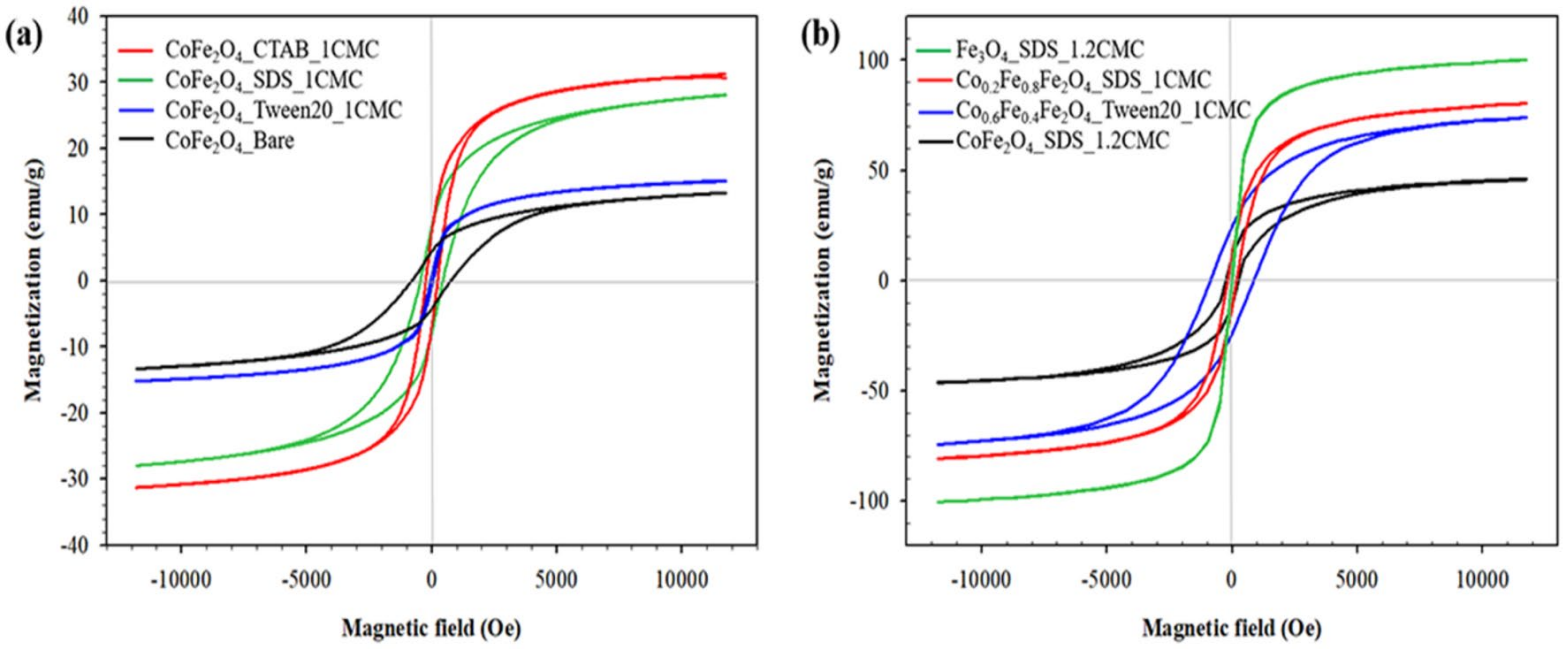
Hysteresis loops of CoFe_2_O_4_ and Co_x_Fe_1−x_Fe_2_O_4_: (**a**) under various surfactant types; (**b**) under various Fe^2+^ contents.

The magnetic properties of cobalt ferrite nanoparticles were measured by the VSM at room temperature (300 K). The saturated magnetization (M_s_), coercivity (H_c_) and magnetic remanence (M_r_) values were obtained from the hysteresis curves in Fig. 8a,b, and are tabulated in Table 2. The hysteresis curves show the large loops of cobalt ferrite nanoparticles with the presence of cobalt atoms namely: CoFe_2_O_4_, Co_0.6_Fe_0.4_Fe_2_O_4_, and Co_0.2_Fe_0.8_Fe_2_O_4_ with the high H_c_ and M_r_ values; thus, the synthesized cobalt ferrite nanoparticles are hard or ferromagnetic materials^45^. On the other hand, the Fe_3_O_4_ hysteresis curve shows the superparamagnetic behavior where the H_c_ and M_r_ values were close to zero^46^.

**Table 2:** Magnetic and electrical properties of Co_x_Fe_1−x_Fe_2_O_4_ nanoparticles.

Sample	VSM			Electrical conductivity (S/cm)	Ref
	M_s_ (emu/g)	H_c_ (Oe)	M_r_ (emu/g)		
CoFe_2_O_4__Bare	13.30	786.66	4.31	1.11 × 10^–2^ ± 9.16 × 10^–4^	This work
CoFe_2_O_4__SDS_1CMC	28.06	448.58	8.18	1.41 × 10^–2^ ± 1.48 × 10^–3^	
CoFe_2_O_4__CTAB_1CMC	31.26	232.52	7.48	1.33 × 10^–2^ ± 1.41 × 10^–3^	
CoFe_2_O_4__Tween20_1CMC	15.15	53.52	1.01	1.13 × 10^–2^ ± 7.07 × 10^–4^	
CoFe_2_O_4__SDS_1.2CMC	46.19	263.02	11.83	2.06 × 10^–2^ ± 9.44 × 10^–5^	
Co_0.6_Fe_0.4_Fe_2_O_4__SDS_1.2CMC	74.19	877.76	24.78	3.94 × 10^–2^ ± 3.03 × 10^–3^	
Co_0.2_Fe_0.8_Fe_2_O_4__SDS_1.2CMC	80.62	190.76	13.56	5.33 × 10^–2^ ± 8.64 × 10^–4^	
Fe_3_O_4__SDS_1.2CMC	100.41	43.03	4.37	1.18 × 10^–1^ ± 1.82 × 10^–2^	
CoFe_2_O_4_	74.08	527.97	23.81	–	^54^
CoFe_2_O_4_	58.40	286.00	12.45	–	^55^
CoFe_2_O_4_	34.70	233.00	47.20	–	^56^
Fe_3_O_4_	63.36	–	–	–	^57^
Fe_3_O_4_	61.92	–	–	–	^58^
Fe_3_O_4_	78.00	–	–	–	^59^
Fe_3_O_4_	87.00	31.00	4.60	9.68 × 10^–3^	^43^

Figure 8a shows the hysteresis curves of CoFe_2_O_4_ as synthesized by various surfactant types. The M_s_ values are 13.30 emu/g, 28.06 emu/g, 31.25 emu/g, and 15.15 emu/g, for the CoFe_2_O_4_ synthesized by using no surfactant, SDS, CTAB and Tween20 with the particle sizes of 42 nm, 16 nm, 20 nm, and 21 nm, respectively. For the CoFe_2_O_4_ as synthesized by SDS and CTAB, it appears that the M_s_ value depends on the particle size, it increases slightly with increasing particle size; a smaller particle has a weaker coordination of surface atoms resulting in a disorder in the surface spins^47^. However, the CoFe_2_O_4_ as synthesized by Tween20 and no surfactant show the lower M_s_ values due to the lower crystallinity^48^, which can be observed from the (311) plane of the XRD patterns in Fig. 2a. The XRD patterns of CoFe_2_O_4_ as synthesized by Tween20 and no surfactant show the weak and broad peaks due to the lower crystallinity relative to the XRD patterns of CoFe_2_O_4_ as synthesized by SDS and CTAB as shown in Fig. 2a.

In the case of Co_x_Fe_1−x_Fe_2_O_4_ as shown in Fig. 8b, the M_s_ values are 46.19 emu/g, 74.19 emu/g, 80.62 emu/g, and 100.41 emu/g for the CoFe_2_O_4_, Co_0.6_Fe_0.4_Fe_2_O_4_, and Co_0.2_Fe_0.8_Fe_2_O_4_, and Fe_3_O_4_, respectively. On comparing with the previous M_s_ values of the bulk CoFe_2_O_4_ (80 emu/g)^49^ and Fe_3_O_4_ (90 emu/g)^47^, the present M_s_ value of Co_x_Fe_1−x_Fe_2_O_4_ increases with increasing Fe^2+^ substitution due to fact that Fe^2+^ provides more unpaired electrons in the 3d orbital leading to the higher number of magnetic moments in the metal ion of the magnetic nanoparticles^50,51^. On comparing the Fe^2+^ and Co^2+^ 3d orbitals, Fe^2+^ has a higher number of unpaired electrons in the 3d orbital resulting in a higher magnetic moment and Bohr magneton which can be approximately by Eq. (7)^45^.

7 $$\upmu_{{\text{s}}} = {\text{g}}\sqrt {{\text{S}}\left( {{\text{S}} + 1} \right)}$$

where μ_s_ is the magnetic moment (Bohr magneton), g is the gyromagnetic ratio or the ratio of the magnetic moment to the angular momentum. For a free electron, g = 2, and S is the sum of the spin quantum numbers where each electron contributes ± 1/2. The S values of Co^2+^ and Fe^2+^ are 3/2 and 4/2, respectively. Thus, the calculated magnetic moments of Co^2+^ and Fe^2+^ are 3.87 magnetons and 4.90 magnetons, respectively. Other previous works also showed the increase of M_s_ values under the substitution of increasing Fe^2+^ in the Co_x_Fe_1−x_Fe_2_O_4_^11,41^.

The H_c_ values of the cobalt ferrite nanoparticles are 263.02 Oe, 877.76 Oe, 190.76 Oe, and 43.03 Oe for CoFe_2_O_4_, Co_0.6_Fe_0.4_Fe_2_O_4_, and Co_0.2_Fe_0.8_Fe_2_O_4_, and Fe_3_O_4_, respectively. Comparing with previous work as shown in Table 2, the H_c_ values of the synthesized CoFe_2_O_4_ and Fe_3_O_4_ are comparable to the previous work. It can be noted that the H_c_ value increases with decreasing x values from 1 to 0.4, along with the increase of the Fe^2+^ mole ratio. Below x value of 0.4, the H_c_ value decreases to the lowest value for Fe_3_O_4_ (x = 0). The result is consistent with the previous work as the highest H_c_ value was found in the case of Co_0.5_Fe_0.5_Fe_2_O_4_ (x = 0.5)^11,52^.

Lastly, it may be noted that the M_s_ values of Fe_3_O_4_ from previous works^43,57–59^ as tabulated in Table 2 were 63.36, 61.92, and 78.00 emu/g, respectively. The presently obtained M_s_ value of Fe_3_O_4__SDS_1.2CMC is 100.41 emu/g which is relatively higher.

### Electrical conductivity of cobalt ferrite nanoparticles

Electrical conductivity of cobalt ferrite nanoparticles was investigated by using a two-point probe meter. The electrical conductivity values of cobalt ferrite nanoparticles are shown in Table 2. For the CoFe_2_O_4_ under various surfactant types, the electrical conductivity values are 1.11 × 10^–2^ S/cm, 1.41 × 10^–2^ S/cm, 1.33 × 10^–2^ S/cm, and 1.13 × 10^–2^ S/cm for the CoFe_2_O_4_ synthesized by using no surfactant, SDS, CTAB and Tween20, respectively. From the electrical conductivity results, CoFe_2_O_4_ can be categorized as a semiconducting material^53^. Under various Fe^2+^ and Co^2+^ substitution, the electrical conductivities are 2.06 × 10^–2^ S/cm, 3.94 × 10^–2^ S/cm, 5.33 × 10^–2^, S/cm and 1.18 × 10^–1^ S/cm for the CoFe_2_O_4_, Co_0.6_Fe_0.4_Fe_2_O_4_, Co_0.2_Fe_0.8_Fe_2_O_4_, and Fe_3_O_4_, respectively. Thus, the electrical conductivity increases with increasing Fe^2+^ mole ratio as shown in Table 2. The electrical conductivity of Fe_3_O_4_ can be attributed to the electron hopping between Fe^3+^ and Fe^2+^ in the octahedral site of the inverse spinel structure. With the substitution of Fe^2+^ by Co^2+^, the electrical conductivity decreases due to the loss of closed-neighbor pairs (Fe^2+^ and Fe^3+^).

## Conclusions

The cobalt ferrite nanoparticles were successfully synthesized by the simple surfactant templated co-precipitation method. The cobalt ferrite nanoparticles show the cubic spinel structure with the nano-sizes varying between 16 and 43 nm with the nearly spherical shapes. The most suitable surfactant for the synthesis of CoFe_2_O_4_ was SDS with the smallest particle size of 16 ± 3 nm. The experimental stoichiometry of cobalt ferrite nanoparticles as obtained by EDS and XPS agreed with the theoretical stoichiometry. The magnetization of cobalt ferrite nanoparticles depended on the size of the nanoparticles and the Fe^2+^ and Co^2+^ ratio. The currently highest magnetization value, M_s_, was obtained from the synthesized Fe_3_O_4_ using the SDS template at 100.41 emu/g. The synthesized Fe_3_O_4_ nanoparticle with high M_s_ is potential to be utilized in various actuator devices and biomedical applications.

## Data Availability

The datasets used and/or analyzed during the current study are available from the corresponding author on reasonable request.
